# Editorial: Maternal-fetal interface: new insight in placenta research, volume II

**DOI:** 10.3389/fendo.2025.1642059

**Published:** 2025-06-26

**Authors:** Cilia Abad, Mariana Farina, Alicia E. Damiano, Reinaldo Marín

**Affiliations:** ^1^ Department of Pharmacology and Toxicology, Faculty of Pharmacy in Hradec Kralove, Charles University, Hradec Kralove, Czechia; ^2^ University of Buenos Aires, Faculty of Medicine, Center of Pharmacological and Botanical Studies [Centro de Estudios Farmacológicos y Botánicos (CEFYBO)-Consejo Nacional de Investigaciones Científicas y Técnicas (CONICET)], Buenos Aires, Argentina; ^3^ University of Buenos Aires, Faculty of Pharmacy and Biochemistry, Department of Biological Science, Buenos Aires, Argentina; ^4^ Consejo Nacional de Investigaciones Científicas y Técnicas (CONICET), University of Buenos Aires, Institute of Physiology and Biophysics Bernardo Houssay 018 (Instituto de Fisiología y Biofísica (IFIBIO) Houssay), Buenos Aires, Argentina; ^5^ Center for Biophysics and Biochemistry (CBB), Venezuelan Institute for Scientific Research (IVIC), Caracas, Venezuela

**Keywords:** placenta, maternal-fetal interface, immune tolerance, cellular invasion, metabolism

The maternal-fetal interface is a dynamic and complex microenvironment where the delicate balance between immune tolerance, cellular invasion, and metabolic regulation determines pregnancy success and the health of both mother and child (1). Recent research continues to unravel the intricate mechanisms at play, revealing new insights into how cellular structures, immune responses, metabolic stress, and environmental exposures converge to shape pregnancy outcomes.

The objective of the Research Topic “*Maternal-Fetal Interface: New Insights in Placenta Research Volume II*” was to bring together original research articles and reviews highlighting recent advances in understanding placental functions and its impact on the health of the mother and her future child. This Research Topic encompasses a diverse range of contributions, including six original articles, a mini-review, three reviews, and a systematic review.

A striking theme emerging from current research is the central role of specialized cellular projections—podosomes and invadopodia—in trophoblast invasion at the maternal-fetal interface (2). These “invasive feet” are crucial for normal placental development, yet their classification remains debated due to the discovery of hybrid structures that blur conventional definitions (2). This conceptual ambiguity highlights the need to re-evaluate how these invasive mechanisms are defined and studied, particularly in the context of pregnancy complications such as placenta accreta and preeclampsia, where trophoblast invasion is either excessive or insufficient.

Epigenetic regulation has become a central focus in understanding the immune microenvironment at the maternal-fetal interface. Mechanisms such as DNA methylation, histone modifications, and non-coding RNA activity are now recognized as crucial for orchestrating the immune balance required for successful implantation and placental development (3). Disruptions in these epigenetic processes have been associated with a range of placenta-related complications, highlighting the potential of targeted interventions that modulate gene expression without altering the underlying DNA sequence (4).

The placenta’s capacity to regulate protein folding and respond to cellular stress is a critical factor in ensuring a healthy pregnancy (Chowdhury et al.). The unfolded protein response (UPR), activated by endoplasmic reticulum (ER) stress, plays a dual role: while a basal level of UPR activity is essential for normal placental development, prolonged or excessive ER stress can lead to cellular dysfunction and adverse pregnancy outcomes (5, 6). This “stress paradox” illustrates the delicate balance between physiological adaptation and pathological disruption, emphasizing the importance of ER homeostasis for both maternal and fetal well-being.

Preeclampsia (PE) remains one of the most formidable challenges in obstetric medicine, due not only to its acute clinical risks but also to its long-term health implications. Recent work suggests that trained innate immunity (TRIM)—a process by which exposure to danger signals during PE epigenetically reprograms maternal immune cells—may provide a mechanistic link between pregnancy complications and future cardiovascular disease (7). This persistent pro-inflammatory state, driven by epigenetic changes, may help explain the elevated risk of hypertension, heart disease, and stroke observed in women with a history of PE. Advancing our understanding of TRIM could open novel avenues for prevention and therapy, both during pregnancy and beyond.

Maternal metabolic status, particularly overweight and obesity, is increasingly recognized as a key modifier of placental and vascular structure (8). Research has demonstrated that maternal overweight is associated with significant remodeling of the umbilical vein, including increased wall thickness and collagen deposition. These structural alterations may compromise the efficiency of nutrient and oxygen delivery to the fetus, potentially influencing long-term health outcomes. Such findings reinforce the importance of optimizing maternal health before and during pregnancy, not only for favorable perinatal outcomes but also for mitigating intergenerational health risks.

Environmental factors are increasingly recognized as significant threats to placental function. Microplastics, once considered as pollutants affecting marine ecosystems, have now been detected in human placental tissue (9). Experimental studies demonstrate that exposure to polystyrene microplastics induces cytotoxicity, oxidative stress, and metabolic disturbances in placental explants, raising serious concerns about their potential to impair fetal development and contribute to pregnancy complications. Given the pervasive presence of microplastics in the environment, further research into their impact on human health is both urgent and essential.

Research into placental oxidative stress has expanded significantly, with growing emphasis on the mechanisms governing antioxidant defenses, gene expression regulation, and the pathophysiology of pregnancy-related diseases (10, 11). Bibliometric analyses reveal a shift toward investigating emerging risk factors, environmental exposures, and the complex interplay between inflammation, stem cells, and immune regulation. These trends reflect a broader understanding that placental health is influenced by an intricate network of genetic, epigenetic, metabolic, and environmental factors.

Advances in reproductive technology, such as preimplantation genetic testing for aneuploidy (PGT-A), are also reshaping our understanding of pregnancy complications (12). Emerging evidence suggests that PGT-A may reduce the risk of preeclampsia in pregnancies involving male fetuses, although this protective effect does not appear to extend to female fetuses. This sex-specific difference underscores the complexity of fetal-maternal interactions and highlights the importance of personalized approaches in fertility treatment and pregnancy management.

Finally, the interaction between inflammation and placental function in the context of maternal diabetes provides valuable insights into the roles of Hofbauer cells, unique and specialized fetal immune cells within the placenta (13). Distinct inflammatory activation profiles and enzyme expression patterns observed in type 1 and gestational diabetes suggest that while immune tolerance is largely preserved, structural and vascular alterations in the placenta may still compromise fetal health. These findings underscore the multifaceted nature of placental adaptation to maternal metabolic and inflammatory states.

Collectively, these studies portray the placenta as both a sentinel and a mediator of maternal and fetal health, finely attuned to a wide array of internal and external signals. The key challenge is to translate these growing molecular and cellular insights into effective interventions capable of preventing or alleviating pregnancy complications, safeguarding maternal health, and ensuring the healthiest possible start for future generations.
